# Ploidy Status of Ovarian Cancer Cell Lines and Their Association with Gene Expression Profiles

**DOI:** 10.3390/biom13010092

**Published:** 2023-01-01

**Authors:** Ming Du, Shuo Zhang, Xiaoxia Liu, Congjian Xu, Xiaoyan Zhang

**Affiliations:** 1 Obstetrics and Gynecology Hospital, Fudan University, Shanghai 200011, China; 2 Shanghai Key Laboratory of Female Reproductive Endocrine Related Diseases, Shanghai 200011, China; 3 Department of Obstetrics and Gynecology of Shanghai Medical School, Fudan University, Shanghai 200032, China

**Keywords:** RNA sequencing, ovarian cancer, polyploid, aneuploid, chromosome alteration

## Abstract

As a cancer type potentially dominated by copy number variations, ovarian cancer shows hyperploid karyotypes and large-scale chromosome alterations, which might be promising biomarkers correlated with tumor metastasis and chemoresistance. Experimental studies have provided more information about the roles of aneuploids and polyploids in ovarian cancer. However, ploidy evaluation of ovarian cancer cell lines is still limited, even in some ploidy-related research. Herein, the ploidy landscape of 51 ovarian cancer cell lines from the Cancer Cell Line Encyclopedia (CCLE) were analyzed, and the ploidy statuses of 13 human ovarian cancer cell lines and 2 murine cell lines were evaluated using G-banding and flow cytometry. Most human ovarian cancer cell lines were aneuploid, with modal numbers of 52–86 and numerical complexity ranging from 5 to 12. A2780, COV434 and TOV21G were screened as diploid cell lines, with a modal number of 46, a low aneuploid score and a near-diploid ploidy value. Two murine cell lines, both OV2944-HM1 and ID-8, were near-tetraploid. Integrated information on karyotypes, aneuploid score and ploidy value supplied references for a nondiploid model construction and a parallel analysis of diploid versus aneuploid. Moreover, the gene expression profiles were compared between diploid and aneuploid cell lines. The functions of differentially expressed genes were mainly enriched in terms of protein function regulation, TGF-β signaling and cell adhesion molecules. Genes downregulated in the aneuploid group were mainly related to metabolism and protein function regulation, and genes upregulated in the aneuploid group were mainly involved in immune regulation. Differentially expressed genes were randomly distributed on all chromosomes, while chromosome 1 alteration might contribute to immune-related alterations in aneuploid cell lines. Chromosome 19 alteration might be potentially significant for aneuploid ovarian cancer cell lines and patients, which needs further verification in ploidy research.

## 1. Introduction

Ovarian cancer is the major cause of death among gynecological cancers. With an insidious onset and rapid progression, approximately 75% of ovarian cancer patients are already at an advanced stage when diagnosed, with less than a 30% five-year survival rate [1,2]. Nearly 80% of ovarian cancers undergo cancer recurrence and acquire chemoresistance, with a low progression-free survival of less than one year for recurrent populations using platinum-based chemotherapy [3,4]. As a hallmark of cancer, approximately 75% of solid tumors show aneuploidy and chromosomal instability (CIN), with a complex and heterogeneous karyotypic landscape [5]. Karyotypic alterations have been considered to contribute to tumor progression and immune editing, shaping the genetic heterogeneity and karyotypic evolution of cancer cells under poor conditions, which directly or indirectly promotes chemoresistance in cancer [6,7,8,9,10]. Focusing on the ploidy status and karyotypic alteration might provide inspiration for overcoming chemoresistance and exploring potentially new therapeutic targets in ovarian cancer.

According to our previous summary of ploidy studies, many observational studies and correlation analyses of aneuploidy and chromosomal instability have been performed in patient samples [11]. Karyotype analysis revealed that nearly 80% of ovarian cancer patients were hyperploid (http://atlasgeneticsoncology.org (accessed on 1 October 2022)). Flow cytometry or image cytometry showed that nondiploid cancer cells existed in approximately 36%~75% of ovarian cancer patients, potentially affecting therapy choice and prognostic prediction [12,13,14]. According to fluorescent in situ hybridization (FISH) and single cell quantitative imaging microscopy (QuantIM), the levels of aneuploidy and CIN increased with drug resistance and tumor relapse in ovarian cancer [15,16]. Copy number variation (CNV) analysis showed that approximately 55% of ovarian cancers had undergone whole genome doubling (WGD) and possessed a ploidy value of 3.3 (not 2.0), with decreased tumor-infiltrating leukocytes (TILs) and increased total mutational burden and cancer occurrence compared with near-diploid cancer patients [17,18,19]. Very recently, hyperploid value of tumor tissues indicated a late stage in high-grade serous ovarian cancer [20]. CNV analysis indicated that hyperploid cells enriched tumor metastasis and chemoresistance in ovarian cancer [21,22,23]. The analysis of cell-free DNA or circulating-tumor DNA supports the value of aneuploidy in the monitoring of tumor progression, therapeutic efficacy and tumor recurrence [24,25]. Overall, as a genetically unstable tumor, aneuploidy and chromosomal instability are prevalent and dynamic in ovarian cancer patients.

Considering the prevalence and significance of karyotypic evolution in ovarian cancer, it is significant to take the ploidy status into consideration in experimental research, which is important for uncovering the underlying mechanisms of ploidy-mediated chemoresistance and exploring ploidy-targeted cancer therapy. The ploidy evaluation of cell lines is the first step in cancer ploidy research because the cell line models are the most basal tools in cancer research. Moreover, a comparison analysis between diploidy and aneuploidy cell lines is also inspiring and can help to explore potential new therapeutic targets based on aneuploid or polyploid cell lines in cancer. Recently, a comparative study based on diploid versus aneuploid cell lines overturned the concept that the knockdown of small GTPase Ran (Ras-related nuclear protein), a potential therapeutic target in cancer, causes cancer cell death but does not affect normal cells [26]. This research demonstrated that Ran knockdown selectively kills aneuploidy rather than diploid ovarian cancer cells. Once again, this study illustrated the significance of ploidy evaluation for cancer cell lines and supplied a new synthetic lethal strategy based on abnormal ploidy status in ovarian cancer.

However, unlike CIN evaluations of patient samples, few basic studies have focused on the ploidy status and chromosomal alteration of ovarian cancer cell lines, and even fewer studies have assessed their basic ploidy status, even though some cell lines are commonly used, which makes current ploidy research in ovarian cancer confusing. Among various ovarian cancer cell lines, a previous CIN study simultaneously evaluated the response to CIN inducers with or without proteasome inhibitors, and tried to uncover the interplay between CIN and sensitivity to proteasome inhibition [27]. However, the basal ploidy and chromosomal status of cancer cell lines were not evaluated at first. Notably, aneuploidy and diploidy cells themselves are significantly different in terms of proteotoxic stress and CIN, which might cause different responses to inducers of CIN or other types of stress [26,28,29,30]. Diploid and aneuploidy cell lines with different levels of CIN were considered in parallel comparisons without being noted, which might have interfered with the results.

Here, we evaluated the ploidy status and chromosomal alterations of 13 commonly used ovarian cancer cell lines for reference and inspiration for further research in ovarian cancer. To better understand the difference in expression profiles between the diploid and aneuploid groups and find potential targets for nondiploid cancer, high throughput RNA-sequencing and enrichment analysis are performed in this study. The effects of chromosome alterations on gene expression were analyzed via chromosome enrichment.

## 2. Materials and Methods

### 2.1. Online Tools

A total of 51 ovarian cancer cell lines from the Cancer Cell Line Encyclopedia (CCLE) and 582 ovarian cancer patients were involved in this study [31]. The aneuploidy score (AS) and ploidy value partially reflect the ploidy status, which were calculated on the basis of a copy number variation (CNV) analysis and a ploidy estimation using the ABSOLUTE algorithm [19,32,33]. AS and ploidy value were obtained from cBioPortal (www.cbioportal.org (accessed on 15 October 2022)). The pathway and process enrichment analyses of genes enriched in specific chromosomes in cell lines and patients were performed using a metascape analysis tool [34].

### 2.2. Cell Lines

A total of 15 cell lines were used for the experiments, including 13 human ovarian cancer cell lines (A2780, A2780CP, SKOV3, Hey, IGROV1, COV434, HO8910, NIHOVCAR3, OVCAR5, OVCAR8, OVISE, TOV21G and TOV112D) and 2 murine cell lines (OV2944-HM1 (HM-1) and ID-8). All cell lines were archived in our laboratory and the HM-1 cell line was a gift from Professor Haiou Liu (Obstetrics and Gynecology Hospital, Fudan University). The cells were cultured in an RPMI-1640 or DMEM or MC5A medium containing 10% fetal bovine serum. All the cell lines were cultured at 37 °C in an incubator with 5% CO_2_.

### 2.3. G-Banding

A karyotype analysis was performed using the classic cytogenetic method [35,36]. The cell lines were harvested for metaphase chromosomes after colcemid treatment (45 ng/mL). After 45 min to 1 hour in colcemid, the cells were incubated for 30 min and placed in a hypotonic solution at 37 °C (KCl solution (75 mM)) followed by a fixation process with methanol-acetic acid (3:1). The chromosomes spreads and G-banding analysis were begun after washing and drying in the drying chamber. A total of 100 metaphase spreads were analyzed to determine the modal numbers and ploidy statuses. The karyotypic analysis was based on the International System for Human Cytogenetic Nomenclature (ISCN) [37]. According to the ISCN, cell lines with a chromosome modal number between 35 and 45 are hypodiploid, and those with a number between 47 and 57 are hyperdiploid; cell lines with a modal number between 58 and 68 are hypotriploid, and those with a number between 70 and 80 are hypertriploid; cell lines with a modal number between 81 and 91 are hypotetraploid, and those with a number between 93 and 103 are hypertetraploid. For numerical complexity, gains existing in at least two metaphases and losses existing in at least three metaphases are considered as clonal numerical alterations [37,38].

### 2.4. Flow Cytometry

Ploidy analysis was performed according to previous analyses [12,13]. The cells were harvested for 4–6 h followed by counting, washing and staining for 30 min using the Cell Cycle Staining Kit (MultiSciences (Lianke) Biotech, Hangzhou, China). A flow cytometric analysis was performed using a Beckman Coulter cytoflex flow cytometer with FACS CytExpert software (Beckman Coulter). A total of 15,000–20,000 events per sample were recorded at the low rate during cell acquisition and DNA analysis. According to the karyotype analysis, the diploid cancer cell line COV434 was mixed (1:1) with other samples to be assessed [39]. The data were analyzed with FlowJo software (Flow Jo, Ashland, OR, USA).

### 2.5. RNA Sequencing

The total RNA was extracted from 6 ovarian cancer cell lines by Trizol reagent (Nuoweizan, Nanjing, China) separately. The RNA quality was checked by an Agilent 2200 (Agilent Technologies, Santa Clara, CA, USA). Only samples with an RIN (RNA integrity number) >7.0 were acceptable for cDNA library construction. The cDNA libraries were constructed using the NEBNext^®^ Ultra™ Directional RNA Library Prep Kit for Illumina (NEB, USA) according to the manufacturer’s instructions. After purification and enrichment, the final cDNA libraries were quantified by an Agilent2200. The tagged cDNA libraries were pooled in equal ratios and used for 150 bp paired-end sequencing in a single lane of the Illumina HiSeqXTen (Illumina, San Diego, CA, USA). The raw data were filtered to remove the adaptor sequences and low-quality reads. The clean reads were then mapped to the human genome (GRCh38_Ensembl104) using the Hisat2 [40]. HTseq was used to obtain the gene counts, and the FPKM method was used to determine the gene expression [41].

### 2.6. Identification of DEGs and Enrichment Analysis

A2780, COV434 and TOV21G were categorized as diploid cell lines and NIHOVCAR3, OVISE, OVCAR8 were categorized as aneuploid cell lines. DESeq2 (V1.6.3) was used to filter the differentially expressed genes (DEGs) among diploid cell lines versus aneuploid cell lines. The DEGs were determined by a log2FC of 1 and an adjusted FDR value of 0.05 [42]. Gene ontology (GO) analysis and pathway analysis via the Kyoto Encyclopedia of Genes and Genomes (KEGG) were performed to determine the biological implications and significant pathways. The top 20 terms of the GO categories and KEGG pathways are shown in the form of bubble maps. Gene Set Enrichment Analysis (GSEA) was also performed. For GSEA analysis, the thresholds for enrichment results were set to FDR < 0.25 and a nominal *p*-value < 0.05.

### 2.7. Statistical Analysis

The difference in ploidy value of 51 cell lines among different pathological types was analyzed with the Kruskal–Wallis Test. Fisher’s exact test was applied to identify the significant GO and KEGG pathways. *p* < 0.05 was considered significant.

## 3. Results

### 3.1. Ploidy Analysis from the CCLE for 51 Ovarian Cancer Cell Lines

**Figure 1:**
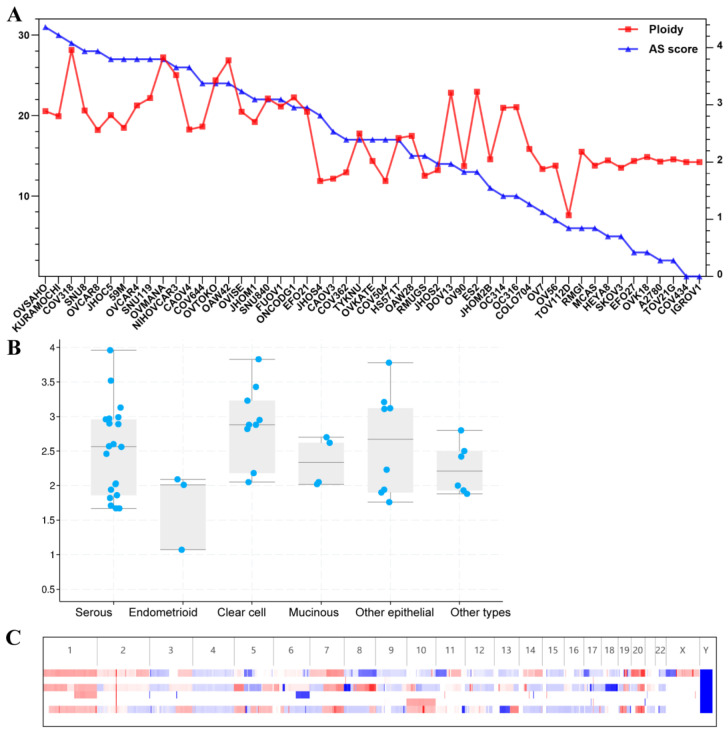
Ploidy and aneuploid score of 51 ovarian cancer cell lines from the CCLE. (**A**): Ploidy and aneuploid score of 51 ovarian cancer cell lines from the CCLE. The aneuploid score and ploidy value are obtained from cBioPortal on the basis of chromosome copy variations and ABSOLUTE estimation. (**B**): Comparison analysis of the ploidy value among different histological types (P = 0.138). Other epithelial: cell lines from cystadenocarcinoma type or adenocarcinoma without clear types; Other types: cell lines from granulosa cell tumor of mixed ovarian carcinoma. (**C**): Copy number variations of representative samples of diploid and aneuploid cell lines. Terms from top to bottom are COV318 (AS = 29, ploidy value = 3.96); COV434 (AS = 0, ploidy value = 2.00); KURAMOCHI (AS = 30, ploidy value = 2.80); A2780 (AS = 2, ploidy value = 2.01); TOV21G (AS = 2, ploidy = 2.05) and OVSAHO (AS = 31, ploidy value = 2.89).

The ploidy statuses of 51 ovarian cancer cell lines were obtained via cBioPortal online analysis, which is based on CNV data and ploidy estimation using the ABSOLUTE algorithm (Figure 1A). Most ovarian cancer cell lines were aneuploid. The ploidy values of nearly all cell lines were not 2.0, and most cell lines possessed relatively high aneuploid scores (Figure 1A). Some cell lines with near-diploid values showed low aneuploid scores, such as SKOV3, A2780, COV434, TOV21G and IGROV1, while some hyperploidy cell lines had high aneuploid scores, such as KURAMOCHI, OVCAR4, OVMANA and NIHOVCAR3. Detailed information on the chromosome alterations and ploidy values is provided in Supplementary Table S1. A comparison analysis suggested that there was no significant difference in ploidy value among the different pathological types (Figure 1B). Compared with cell lines showing both relatively high ploidy values and relatively high aneuploid scores, cell lines with low ploidy values and low aneuploid scores showed fewer copy number variations (Figure 1C).

### 3.2. Karyotype Numerical Complexity and Integrated Ploidy Information for Ovarian Cancer Cell Lines

**Figure 2:**
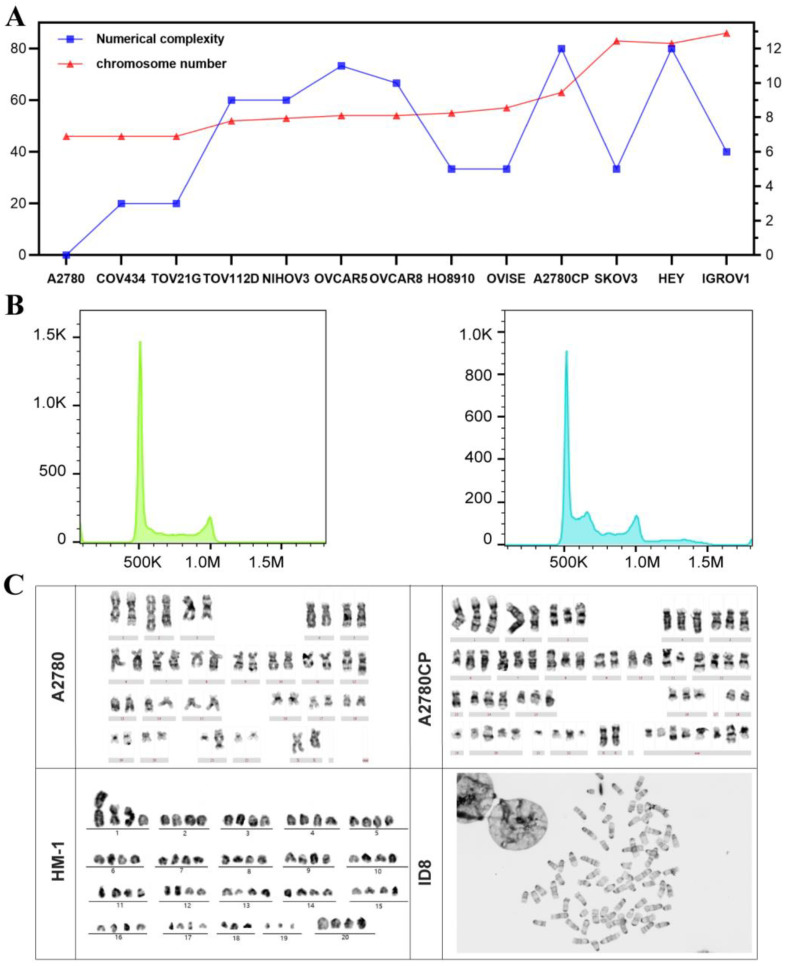
Karyotype and ploidy analysis of 13 commonly used ovarian cancer cell lines. (**A**): Chromosome modal number and numerical complexity of 13 human ovarian cancer cell lines. (**B**): Ploidy analysis of cell lines using flow cytometry. (Left panel: diploid cell line COV434; Right panel: COV434 mixed with SKOV3 in a ratio of 1:1). (**C**): Representative figures of karyotype analysis of human diploid and aneuploid cell lines and murine cell lines.

FCM and G-banding were performed to assess the karyotypes and chromosome numerations of 13 commonly used human cell lines and 2 murine cell lines. According to the karyotype analysis using G-banding, only three ovarian cancer cell lines, A2780, TOV21G and COV434, were diploid cell lines with a modal number of 46 and karyotypical stability with relatively low numerical complexity (Figure 2A). The other human cell lines were aneuploidy and showed relatively large-scale numerical changes (Figure 2A and Table 1). The karyotype details showed that chromosomes 2, 7, 9, 17, 18, 20, 21 and X are commonly altered in human aneuploid cell lines (Supplementary Table S1).

However, some tetraploid cell lines presented low numerical complexity, such as SKOV3 and IGROV1. The ploidy analysis also revealed a significant difference between diploid and aneuploid cells using FCM, consistent with the karyotype analysis (Figure 2B). Most human cell lines had various structural chromosome changes and showed multiple and clonal alterations, which are manifested as chromosomal additions, arrangements, deletions and the presence of marker chromosomes (Figure 2C). Two murine cell lines, both HM-1 and ID-8, were near-tetraploid, with modal number of 78 and 73, respectively (Figure 2D). For a more comprehensive evaluation of ploidy status, the chromosome number, aneuploid score and ploidy value were collected from cell lines with all three parameters in Table 2.

### 3.3. Difference in Expression Profiles between Human Diploid and Aneuploid Ovarian Cancer Cell Lines

**Figure 3:**
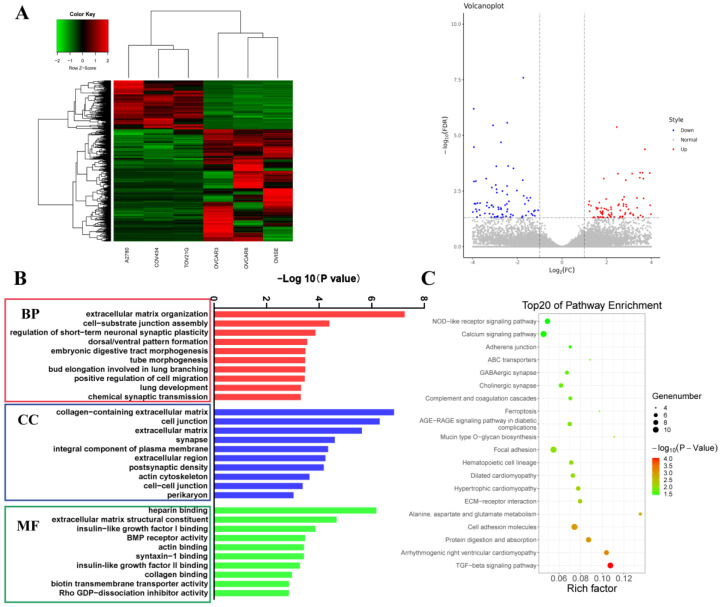
Identification and functional analysis of the differentially expressed genes. (**A**): Heat map and volcano plot of the differentially expressed genes (DEGs) among diploid cell lines versus aneuploid cell lines. (**B**): Gene Ontology summary of DEGs for biological process categories (BP), cell component categories (CC) and molecular function categories (MF). (**C**): Kyoto Encyclopedia of Genes and Genomes summary of DEGs.

Considering the three parameters in Table 2, three cell lines with a modal number of 46, a ploidy value of ±2.0 and a low aneuploid score were categorized into diploid cell lines according to the integrated ploidy information, including A2780, COV434 and TOV21G. NIHOVCAR3, OVISE and OVCAR8 were categorized into the aneuploid group due to their modal number, relatively high ploidy value and relatively high aneuploid score. Pathological types were not taken into consideration due to their nonsignificant association with ploidy value. RNA sequencing and comparison analysis were performed between the diploid and aneuploid groups to explore ploidy control and regulation. Compared with the diploid cells, 419 genes were upregulated and 182 genes were downregulated in the aneuploid cells (Figure 3A). To understand the biological implications of the differentially expressed genes (DEGs), Gene Ontology (GO) analysis and Kyoto Encyclopedia of Genes and Genomes (KEGG) analysis were carried out. According to the top 15 significant terms in BP (biological process), CC (cell component) and MF (molecular function) from the GO enrichment analysis, the DEGs between the diploid and aneuploid groups were mainly involved in the following pathways: “positive regulation of transcription by RNA polymerase II”, “extracellular matrix organization”, “chemical synaptic transmission” and “response to ATP” (Figure 3B and Supplementary Materials). KEGG analysis suggested that these DEGs were particularly enriched in the “NOD-like receptor signaling”, “TGC-β signaling”, “protein digestion and absorption” and “focal adhesion” pathways (Figure 3C).

**Figure 4:**
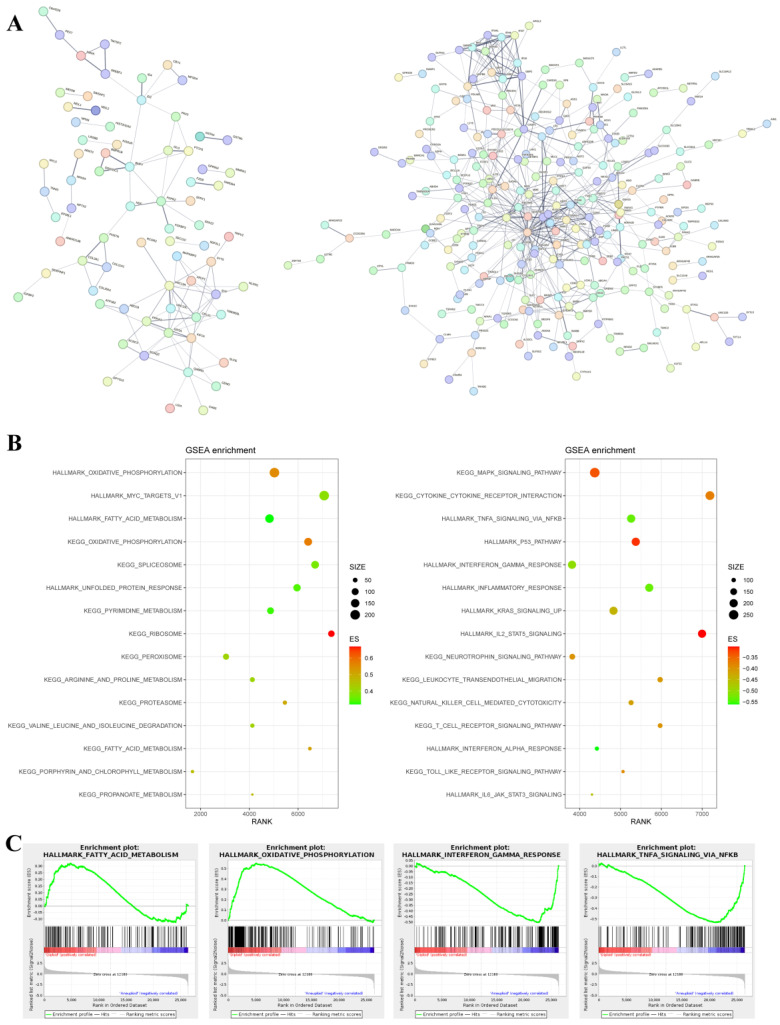
GSEA of the differentially expressed genes. (**A**): Protein-protein interaction enrichment analysis of genes downregulated (**left**) and upregulated (**right**) in aneuploid cell lines. (**B**): GSEA summary of genes downregulated (**left**) and upregulated (**right**) in aneuploid cell lines. (**C**): Significant gene sets (left two panels: downregulated genes in aneuploid group; right two panels: upregulated genes in aneuploid group).

A protein–protein interaction (PPI) enrichment analysis showed some interactions among the downregulated and upregulated genes in aneuploid cell lines (Figure 4A). A gene set enrichment analysis (GSEA) showed that genes downregulated in the aneuploid group were mainly enriched in metabolism-related gene sets, such as “ribosome”, “hallmark oxidative phosphorylation”, “hallmark fatty acid metabolism” and “KEGG arginine and proline metabolism” (Figure 4B,C). Genes upregulated in the aneuploid group were mainly enriched in immune-related pathways, such as the “KEGG NOD-like receptor signaling pathway”, “hallmark interferon gamma response”, “KEGG cytokine-cytokine receptor interaction”, “hallmark inflammatory response”, and “hallmark TNFA signaling via NFκB” (Figure 4B,C).

### 3.4. Effects of Chromosome Alterations on Gene Expression Regulation

**Figure 5:**
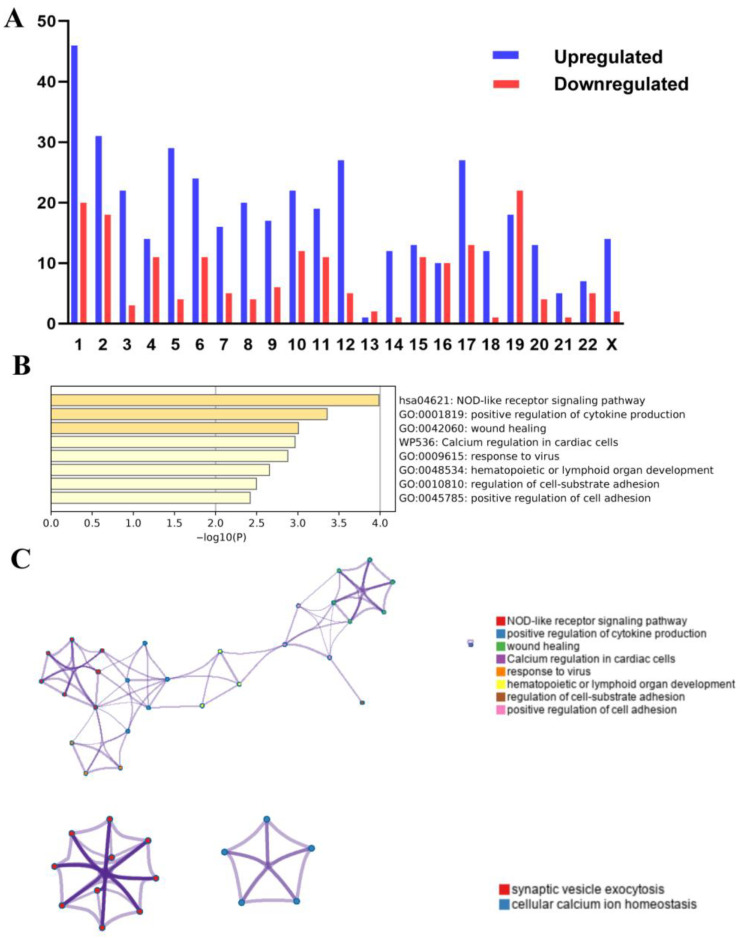
Location of differentially expressed genes and their functional analysis. (**A**): The enrichment of chromosomes using 419 upregulated DEGs and 182 downregulated DEGs in aneuploid cell lines. (**B**): Enrichment using the 46 upregulated DEGs on chromosome 1. (**C**): Enrichment using the 46 upregulated DEGs on chromosome 1 (**left**) and the 22 downregulated DEGs on chromosome 19 (**right**).

Chromosome 1 (chr1) and chromosome 19 (chr 19) alterations are among the most frequent cytogenetic changes in epithelial ovarian cancer [43,44,45,46]. We tried to determine whether the 419 DEGs upregulated and 182 genes downregulated in aneuploid ovarian cancer cell lines may result from the chr1 and chr19 alterations. According to the chromosome location, all the DEGs were randomly distributed across all chromosomes, but the majority were found on chromosomes 1 (66 genes, 11%), 2 (49 genes, 18%), 17 (40 genes, 6.7%) and 19 (40 genes, 6.17%). However, chromosome 1 contained the most up-regulated genes (46 genes, 11%) and chromosome 19 contained the most downregulated genes (22 genes, 12%) (Figure 5A). The enrichment analysis showed that the 46 upregulated genes were most significantly enriched in the immune-related pathways, such as the “KEGG NOD-like receptor signaling” and “positive regulation of cytokine production” pathways (Figure 5B,C). The 22 downregulated genes were most significantly enriched in the “BP-synaptic vesicle exocytosis”, “export from cell” and “synapse organization” pathways (Figure 5C). The results indicated that chr1 and chr19 alteration might mainly contribute to the expression changes related to immunity and exocytosis in aneuploid cell lines.

### 3.5. Genomic Alteration Analysis between Euploid and Aneuploid Ovarian Cancer Patients

**Figure 6:**
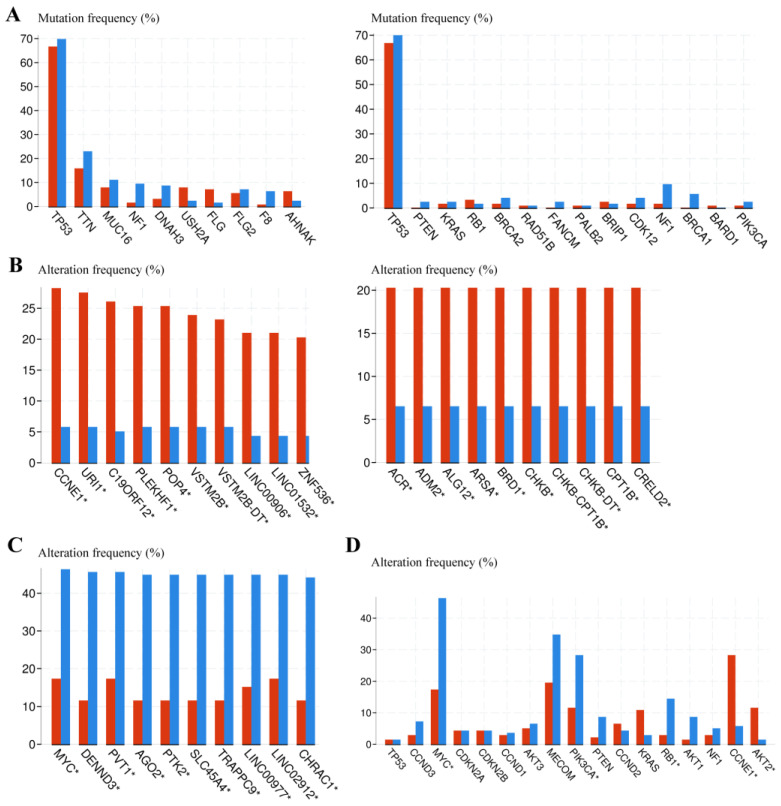
Comparison of genetic alterations between near-euploid and highly-aneuploid ovarian cancer patients. (**A**) Mutation frequency in highly-aneuploid (high-aneuploid score) group and near-euploid (low-aneuploid score) group. Left panel: Gene mutations with the highest frequency in any group; Right panel: Common mutations in serous ovarian cancer. (Blue: euploid group; red: aneuploid group). (**B**) Copy number alterations enriched in the highly-aneuploid group. Left: amplification; right: deletion. (**C**) Copy number alterations enriched in the near-euploid group. (**D**) Copy number alterations of genes frequently altered in serous ovarian cancer.

**Figure 7:**
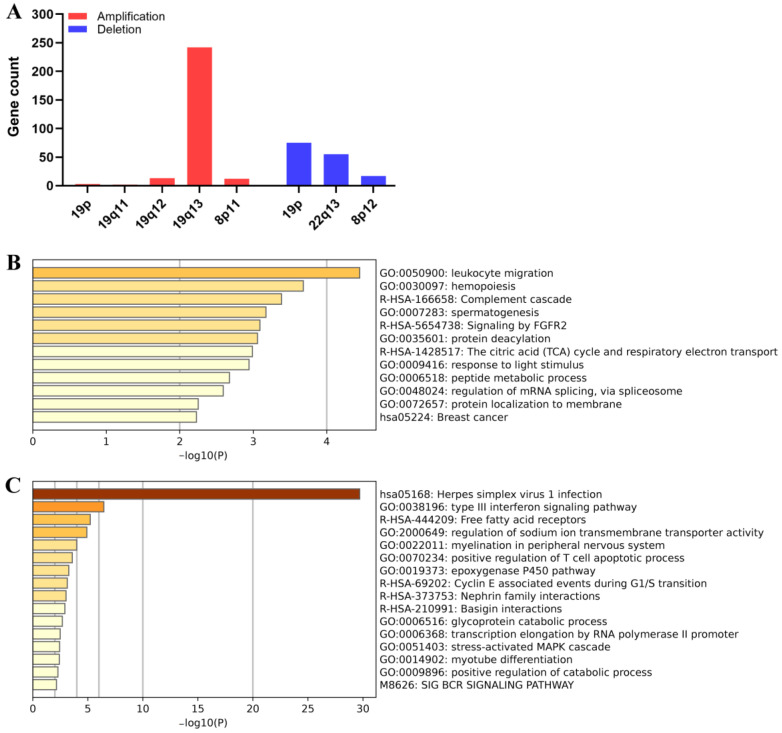
Chromosome location and enrichment analysis of genes with significant CNV in the highly-aneuploid group. (**A**) Chromosome location of genes with CNV enriched in the highly-aneuploid group. (**B**) Enrichment analysis of genes with significant deletions enriched in the highly-aneuploid group. (**C**) Enrichment analysis of genes with significant amplifications enriched in the near-euploid group.

To better understand the association between potential crucial chromosome alterations and ploidy status in ovarian cancer patients, we analyzed the genomic alteration between euploid and aneuploid ovarian cancer patients. A total of 582 ovarian cancer patients were included and ranked by aneuploid score from highest to lowest, which partially reflects the aneuploid level [33]. The top quarter were categorized as a highly-aneuploid group and the bottom quarter were categorized as a near-euploid group, similar to previous classifications of cell lines [32]. The genomic alterations were analyzed between the highly aneuploid and near-euploid group using the cBioPortal online tool. There was no significant difference between the two groups in the frequency of the common mutations in ovarian cancer [20] or the mutations with the highest frequency in any group (Figure 6A). However, the copy number variations (CNV) between the two groups showed a clear difference (Figure 6B,C). The frequency of CNV in frequently altered genes in serous ovarian cancer was also significantly different between the two groups (Figure 6D) [42]. The location of genes with CNV significantly enriched in the highly-aneuploid group showed that these genes are mostly enriched in chromosome 19, regardless of amplifications or deletions (Figure 7A). An enrichment analysis showed that these genes in chromosome 19 were mainly related to the immune function (Figure 7B). All these data showed that the chromosome 19 alteration might be potentially significant for aneuploid ovarian cancer.

## 4. Discussion

In this study we evaluated the karyotype and chromosomal numerical complexity of 13 human ovarian cancer cell lines and 2 murine cell lines using G-banding and flow cytometry. According to the integrated ploidy status based on three parameters, modal number, ploidy value and aneuploid score, three relatively stable diploid cell lines, A2780, COV434 and TOV21G, were screened in our study and can be used for nondiploid model construction for further aneuploidy and polyploidy research in ovarian cancer. However, most commonly used human and murine ovarian cancer cell lines are aneuploid or hyperploid, showing a relatively high ploidy value and high aneuploid score and large-scale numerical changes.

Many ploidy studies have used the diploid colon cancer cell line HCT116 and the non-transformed diploid epithelial cell line RPE-1 to construct paired aneuploid and polyploid cell models [17,32,47]. However, in ovarian cancer ploidy research, the pre-evaluation of the basal ploidy status of cancer cell lines is usually ignored before the construction of nondiploid cell models. For example, the commonly used cell lines for the construction of the polyploidy giant cancer cells (PGCCs) Hey and SKOV3 [48] are already near-polyploidy. Herein, the three diploid cell lines screened in our study provide alterations for further model construction in ploidy research in ovarian cancer.

In addition to supplying diploid alterations for model construction, the ploidy status of commonly used cell lines provides inspiration for future comparative analyses of diploid and aneuploid cell lines in ovarian cancer. Nondiploid cells are different from diploid cells in terms of cell fitness and characteristics, featuring worse genomic, metabolic, proteotoxic stress and immune stress, manifested as senescence and cell cycle arrest [49,50]. Through comparisons of diploid and aneuploid cell lines at the same time, it might be possible to uncover mechanisms of current therapy that already exist and find new therapeutic targets in ovarian cancer. Additionally, nondiploid cell models from diploid parentals can be pooled with aneuploid cell lines in the parallel comparisons of the responses of genetic and chemical regulations.

Some commonly used chemotherapy drugs are not selectively lethal for cancer cells and usually cause the death of normal cells with rapid reproduction, such as carboplatin and paclitaxel. A previous study revealed that these two drugs showed no selectivity between diploid and aneuploid cells, although most cancers are highly aneuploidy [51,52,53]. The prevalent characteristics in cancer, aneuploidy and polyploidy, as unique features of cancer cells compared with diploid normal cells, have already been adopted in the precise identification of circulating tumor cells (CTC) [54,55,56]. However, this feature has not been well adopted as a therapeutic target in potential ploidy-selective cancer therapy. Comparisons between nondiploid and diploid cell lines are necessary to explore ploidy-selective targets and reduce ploidy-related chemoresistance.

A comparative analysis of expression profiles showed that, compared with diploid cell lines, genes related to oxidative phosphorylation, lipid metabolism and regulation of protein function were downregulated in the aneuploid group. Consistent with the survival stress reported in nondiploid cells [11], metabolism and protein regulation might be prominent vulnerabilities for aneuploid cell lines, a possibility that needs further verification. Genes related to the immune response were significantly upregulated in aneuploidy cell lines, especially the NOD-like signaling pathway, NF-κB signaling pathway, IFN-α or IFN-γ responses and the inflammatory response. According to The Cancer Genome Atlas (TCGA), aneuploidy and polyploidy correlated with immune evasion, with the downregulation of genes related to antigen presentation, the lower infiltration of CD8+ T cells and NK cells, and the upregulation of immune evasion pathways in nondiploid cancer [10,33,57,58]. Acute aneuploidy induced by ploidy shifts can increase the clearance of NK cells, and tetraploid cancer cells have difficulty forming tumors in immunocompetent mice [59,60]. Overall, currently, the nondiploid-immune association remains poorly understood. Our cell line analysis showed that aneuploidy might be tightly related to the immune response and future work should concentrate more on the ploidy-immune association, which might provide new insights for cancer immunotherapy. Moreover, chromosome 1 alteration might contribute to the alterations in immune-related function, which needs more evidence in cell lines. The location analysis showed that chromosome 1 and chromosome 19 alteration might be significant for aneuploid cell lines and aneuploid ovarian patients, which needs further verification.

For the integrated evaluation of ploidy status, it might be more comprehensive to combine cytogenetic methods and sequence or array data to obtain the overall and detailed chromosome variations. In our research, the cytological data of SKOV3 and IGROV1 indicated that they are near-tetraploid, but the ABSOLUTE estimation showed that the ploidy status was 1.9 and 2.0, respectively. The discordance of the ploidy evaluation results might be due to the difference between the two methods. The aneuploid score or the ploidy value using ABSOLUE estimation is based on copy number variations but cannot fully reflect cytogenetics [19]. Although the karyotypes of IGROV1 and SKOV3 are near-tetraploid, they have less CIN, showing less CNV and less numerical complexity (Table 1 and Table 2). This reminds us that the ploidy estimation according to copy number variations is not exactly same as the ploidy value according to the G-banding karyotype. Despite the relevance of ABSOLUTE estimation, G-banding is still needed for the ploidy evaluation of cell lines. Combined technologies and the integrated evaluation of chromosome alterations are necessary for ploidy evaluation in cancer cell lines [61].

The largest limitation of this study is that the functional regulation of differentially expressed genes was not verified; more work will be needed for clarification in ploidy research. Additionally, the number of ovarian cancer cell lines used in our study was limited. Despite these limitations, the ploidy information and comparison analysis in this study still provide inspiration for ploidy research in ovarian cancer.

## 5. Conclusions

In conclusion, we analyzed the ploidy status of 51 ovarian cancer cell lines using the cBioPortal and evaluated the karyotype numerical complexity of 13 commonly used human ovarian cell lines and 2 murine cell lines using G-banding and flow cytometry. We explored the biological function of differentially expressed genes between a diploid and aneuploid group categorized by modal number, ploidy value and aneuploid score. Inflammation- and immune-related genes were overexpressed in the aneuploidy group while metabolism- and protein-related genes were downregulated in aneuploid cell lines. Chromosome enrichment suggested that chromosome 1 and chromosome 19 alteration might contribute to ploidy-related alterations in gene expression. In future research, on the basis of the heterogeneous ploidy status of cell lines, more efforts need to be made to uncover ploidy-mediated chemoresistance and explore ploidy-targeted cancer therapy in further ploidy studies in ovarian cancer.

## Figures and Tables

**Table 1:** Karyotype analysis of 13 commonly used ovarian cancer cell lines.

Histological Type	Name	Karyotype Analysis		
		Modal Number	Ploidy	Numerical Range ^1^
Adenocarcinoma				
Serous	NIHOVCAR3	53	hyperdiploid	49–57
	OVCAR5	54	hyperdiploid	47–57
	OVCAR8	54	hyperdiploid	50–59
Endometrial	TOV112D	52	hyperdiploid	47–55
	A2780	46	diploid	46
	A2780CP	63	hypotriploid	56–67
Clear cell	OVISE	57	hypotriploid	54–58
	TOV21G	46	diploid	45–47
Mixed	IGROV1	86	hypotetraploid	84–89
NS ^2^	HO8910	55	hyperdiploid	51–55 ^3^
	HEY	82	hypotetraploid	72–83
Cystadenocarcinoma	SKOV3	83	hypotetraploid	79–83
Granulosa cell tumor	COV434	46	diploid	44–46

^1^ Only clonal numerical alterations were taken into the analysis. ^2^ Cell lines with unclear detailed subtypes of adenocarcinoma. ^3^ Approximately 20% of metaphases are near-tetraploid for this cell line, and the range number is between 98 and 101 in the near-tetraploid subpopulation.

**Table 2:** Integrated information on the ploidy status of 9 commonly used ovarian cancer cell lines.

Name	Ploidy Status			
	Modal Number	Ploidy Estimation	Aneuploid Score	Ploidy Value
IGROV1	86	hypotetraploid	0	2.00
SKOV3	81	hypotetraploid	5	1.90
OVISE	57	hypotriploid	23	2.88
OVCAR8	54	hyperdiploid	28	2.56
NIHOVCAR3	53	hyperdiploid	26	3.52
TOV112D	52	hyperdiploid	6	1.07
A2780	46	diploid	2	2.01
TOV21G	46	diploid	2	2.05
COV434	46	diploid	0	2.00

## Data Availability

Ploidy data and aneuploid scores can be obtained from the cBioPortal website (www.cbioportal.org (accessed on 15 October 2022)).

## Supplementary Materials

The following supporting information can be downloaded at: https://www.mdpi.com/article/10.3390/biom13010092/s1, Table S1: Ploidy status of ovarian cancer cell lines.
